# Effects of and satisfaction with short message service reminders for patient medication adherence: a randomized controlled study

**DOI:** 10.1186/1472-6947-13-127

**Published:** 2013-11-16

**Authors:** Hsiu-Ling Huang, Yu-Chuan Jack Li, Yueh-Ching Chou, Yow-Wen Hsieh, Frank Kuo, Wen-Chen Tsai, Sinkuo Daniel Chai, Blossom Yen-Ju Lin, Pei-Tseng Kung, Chia-Jung Chuang

**Affiliations:** 1 Department of Health Services Administration, China Medical University, 91, Hsueh-Shih Road, Taichung 40402, Taiwan; 2 Hospital Administration Commission, Department of Health, Executive Yuan, Taipei, Taiwan; 3 Department of Graduate Institute of Biomedical Informatics, Taipei Medical University, Taipei, Taiwan; 4 Department of Pharmaceutical Service, Taipei Veterans General Hospital, Taipei, Taiwan; 5 Department of Pharmaceutical Service, China Medical University Hospital, Taichung, Taiwan; 6 Department of Information Management, National Taiwan University, Taipei, Taiwan; 7 Department of Healthcare Administration, Asia University, Taichung, Taiwan

**Keywords:** Short message service (SMS), Medication reminders, Personal medication platform, Patient compliance

## Abstract

**Background:**

Medication adherence is critical for patient treatment. This study involved evaluating how implementing Short Message Service (SMS) reminders affected patient medication adherence and related factors.

**Methods:**

We used a structured questionnaire to survey outpatients at three medical centers. Patients aged 20 years and older who were prescribed more than 7 days of a prescription medication were randomized into SMS intervention or control groups. The intervention group received daily messages reminding them of aspects regarding taking their medication; the control group received no messages. A phone follow-up was performed to assess outcomes after 8 days. Data were collected from 763 participants in the intervention group and 435 participants in the control group.

**Results:**

After participants in the intervention group received SMS reminders to take medication or those in the control group received no messages, incidences of delayed doses were decreased by 46.4 and 78.8% for those in the control and intervention groups, respectively. The rate of missed doses was decreased by 90.1% for participants in the intervention group and 61.1% for those in the control group. We applied logistic regression analysis and determined that participants in the intervention group had a 3.2-fold higher probability of having a decrease in delayed doses compared with participants in the control group. Participants in the intervention group also showed a 2.2-fold higher probability of having a decrease in missed doses compared with participants in the control group.

**Conclusions:**

Use of SMS significantly affected the rates of taking medicine on schedule. Therefore, daily SMS could be useful for reminding patients to take their medicine on schedule.

## Background

Poorly treated chronic diseases both increase health care costs and reduce patient quality of life [1]. A report published by the World Health Organization (WHO) in 2003 indicated that effective and innovative strategies for improving medication adherence can more significantly influence human health compared with advancements in medical techniques [2].

Patients often forget or delay their consumption of medication or neglect the instructions of healthcare providers. According to a literature review, an average of 48–80% of patients with chronic psychiatric diseases adhered to their prescribed treatment [3]. An average of 25% of diabetes patients and 53% of hypertensive patients adhered to their prescribed treatment for 6 months [4]. Such low medication adherence by various patient groups with chronic diseases has compelled the worldwide medical community to increasingly focus on applying technology to remedy this situation.

Regarding case management, using phone follow-ups improves the clinical symptoms of patients, facilitating the early identification of complications and enhancing patient adoption of healthy lifestyles [5]. The WHO recommended implementing an innovative service model not limited to face-to-face services to manage chronic diseases; they recommended that cell phones be used to provide timely services [6]. In developed countries, healthcare institutions often use communication technology to remind patients of follow-up appointments, generating a positive image of the healthcare institution and strengthening patient loyalty. Text messaging, also known as Short Message Service (SMS), is a simple and cost-effective tool for providing medication reminders that has been employed by several healthcare services [7,8]. Studies have noted positive changes when an SMS reminder was used to increase adherence to treatment programs [8-10]. Other studies showed positive effects on health-related behaviors regarding SMS interventions [1,11].

We studied the effectiveness of SMS reminders as an intervention to determine whether they improved patient medication adherence. The primary aim was to determine how SMS medication reminders reduced delayed and missed medications doses; the secondary aim included determining patient satisfaction with and demand for SMS-based interventions.

## Methods

### Study design

The study was conducted between November 1, 2010 and October 31, 2011. After we obtained approval from the Institutional Review Boards of the study hospitals (T.V.G.H., 201006021IC; C.M.U.H., 98–08–01A; and W.H., 100008), hospital pharmacists randomly assigned two patients to the intervention group and one to the control group when patients met the inclusion criteria. We used the systematic sampling method to assign qualified patients to each group. Odd-numbered or even-numbered patients from the pharmacy department registration were assigned to each group. Subsequently, trained interviewers conducted one-on-one interviews with the potential participants, explaining the research purpose and methods in outpatient settings such as the outpatient dispensary waiting area. After agreeing to participate in the study, the patients completed a questionnaire-based pretest.

**Figure 1:**
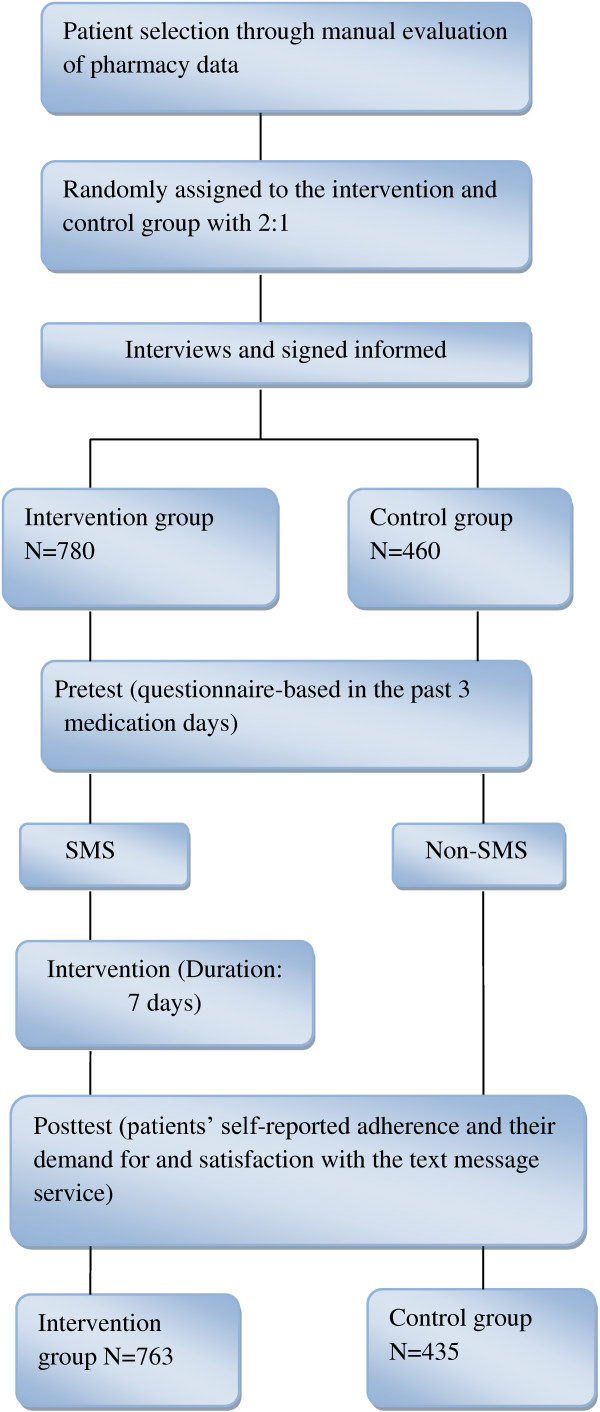
The SMS study screening flowchart.

Patients in the intervention group received SMS reminders reminding them to take their medication at specific times for 7 days beginning on the second day of enrollment, and could not choose the times the reminders were sent. On the eighth day after completing the intervention we conducted a phone-based survey to assess patient medication compliance and demand for and satisfaction with the “text message medication reminder service.” Figure 1 shows the study screening flowchart.

### Participants

Inclusion criteria for participants were: (a) literate patients aged 20 years or older able to communicate with the investigators; (b) having a prescription longer than 7 days; (c) possessing a cell phone and knowing how to receive text messages; (d) and having at least one medication nonadherence (delayed or missed dose) experience in the past 3 days of taking medicine.

We recruited participants from three medical centers based on the large outpatient volume in each hospital. Because we focused on the effect of using SMS in the intervention group, we deliberately increased the sample size of this group. The sample sizes of the intervention and control groups were allocated at a ratio of 2:1.

### Intervention

After patients signed and submitted informed consent forms, they completed a questionnaire-based pretest. We then collected patients’ cell phone numbers to facilitate convenient post-test phone interviews. Patients’ identities were recognized using hospital information system (HIS) databases; their prescriptions were obtained from the clinical department they visited during recruitment and exported to the SMS system for sending reminders. Beginning on the following day, medication reminders were sent to participants by using the SMS system; text messages were sent only to participants in the intervention group.

The SMS content depended on participants’ medication and frequency of medication use. If a participant used multiple medications, he or she received reminders for each. The messages comprised varying content such as visitation date, hospital name, medication name, dosing frequency, dose, and administration methods. The first message contained complete medication information. For example, “A warm reminder! You visited the xx clinic at xx Hospital on 2010/3/15. We would like to remind you to take your prescription, which includes Panadol® (one tablet per day for 7 days), Lasix® (0.5 tablet per day immediately following a meal for 7 days), and Lontex® (0.5 tablet once per day for 7 days). Please remember to take this medication on time. We wish you a prompt recovery.” The second and subsequent messages contained only simple medication reminders.

### Measurements

The research tools comprised an investigator-designed questionnaire that involved a pretest and post-test. The pretest comprised basic demographic questions (e.g., sex, age, and education level), medical conditions, and self-reported medication use experiences (frequency of daily medication use, delayed doses, or missed doses during the previous 3 days). If participants did not take medicine and passed more than a half-period of time between two doses, we defined it as a “missed dose.” For example, if participants needed to take medicine twice a day (i.e., once every 12 hours), and took medicine more than 6 hours late, it qualified as a missed dose. If participants did not take medication as prescribed regarding timing or dosage, but took the medicine within 6 hours, we defined it as a “delayed dose.”

The post-test questionnaire was conducted by phone interview on the eighth day after the study. The post-test questions comprised structured items for the participants’ self-reported adherence to medication during the previous 3 days and their demand for and satisfaction with the SMS medication reminder service. Open-ended questions were also included to obtain participants’ recommendations regarding the service. The interviewers also asked the participants whether they would recommend the service to their family or friends.

To verify the validity of the questionnaire, five experts performed a content validity test, evaluating whether the questionnaire items conformed to the study topic. The average content validity index (CVI) obtained was 0.9. Regarding reliability, the Cronbach’s α coefficient for internal consistency reliability was 0.9, indicating good questionnaire reliability.

### Data analysis

We used SAS statistical software to manage and analyze the data obtained from the questionnaires and descriptive statistics to determine the frequency, percentage, and mean values of each demographic variable. Regarding inferential statistics, we performed chi-square tests to identify differences between the groups regarding improvements in medication adherence and the demand for and satisfaction with the SMS medication reminder service. We used McNemar’s test to compare the pretest and post-test data within each group to examine distinct decreases in the incidence of delayed or missed medication doses before and after the intervention. Because the number of participants recruited from the three study hospitals differed and the management context and service model of the hospital at which most participants were recruited could affect the results of the intervention, we used weighted logistic regression to examine the factors that influenced improvements in the incidence of delayed or missed doses. We used odds ratios (OR) to describe the effect sizes.

## Results

### Baseline data

The intervention group and control group comprised 780 and 460 participants at the time of the pretest, respectively. The number of valid participants who completed both surveys was 763 in the intervention group and 435 in the control group. We obtained 1,198 total questionnaires from the three study hospitals. In each hospital, we collected 188, 225, and 350 questionnaires for the intervention group and 117, 118, and 200 questionnaires for the control group.

We collected data related to the patient medication and basic demographic characteristics such as sex, age, and education level (Table 1). According to the pretest results, 88.1% of the patients in the control group reported infrequently missing a medication dose, compared with 88.5% of patients in the intervention group. The proportion of patients who previously delayed a dose was 80.2 and 84.7% for the control and intervention groups, respectively. The proportion of patients who previously missed a dose was 43.7 and 46.1%, for the control and intervention groups, respectively. The pretest showed no significant differences regarding medication use experience between the groups (P > 0.05). Regarding basic demographic characteristics, sex, age, and living status did not significantly differ between the groups (P > 0.05). Regarding chronic diseases, heart disease, diabetes, and hypertension were the most common conditions for patients in both groups. Most of the chronic diseases listed in Table 1 demonstrated similar distribution in both groups.

**Table 1:** Descriptive statistics of patient demographics and personal information

**Variable**	**Control group**		**Intervention group**		**p-value**
	**N = 435**	**%**	**N = 763**	**%**	
**Medication adherence history**					
**Have you ever NOT adhered to medication prescriptions?**					0.828
Seldom	383	88.1	675	88.5	
Often	52	12.0	88	11.5	
**Delayed doses**					
No	86	19.8	117	15.3	
Yes	349	80.2	646	84.7	
**Missed doses**					
No	245	56.3	411	53.9	
Yes	190	43.7	352	46.13	
**Socioeconomic status**					
Gender					0.574
Male	211	48.5	383	50.2	
Female	224	51.5	380	49.8	
Age (years)					0.085
20 ~ 34	96	22.1	162	21.2	
35 ~ 49	135	31.0	227	29.8	
50 ~ 64	146	33.6	229	30.0	
≧ 65	58	13.3	145	19.0	
Education level					< .001
≧ Elementary	27	6.2	34	4.5	
Junior High School	52	12.0	65	8.5	
High School	89	20.2	275	36.0	
College	91	20.9	151	19.8	
University	135	31.0	179	23.5	
Graduate school or above	41	9.4	59	7.7	
Occupation					0.026
Unemployed	26	6.0	22	2.9	
Military	1	0.2	3	0.4	
Civil servant	45	10.3	57	7.5	
Teacher	33	7.6	49	6.4	
Student	29	6.7	38	5.0	
Housekeeper	60	13.8	103	13.1	
Self-employed	67	15.4	116	15.2	
Medicine	10	2.3	46	6.0	
Retired	33	7.6	78	10.2	
Freelancer	16	3.7	37	4.9	
Services	57	13.1	103	13.5	
Worker	47	10.8	95	12.5	
A.F.F.H.	3	0.7	2	0.3	
Other	8	1.8	14	1.8	
**Variable**	**Control group**		**Intervention group**		**p-value**
	**N = 435**	**%**	**N = 763**	**%**	
Average monthly household income					0.025
≦ 30,000 NTD	94	21.6	122	16.0	
30,001-60,000 NTD	110	25.3	193	25.3	
60,001-90,000 NTD	93	21.4	152	19.9	
90,001-120,000 NTD	34	7.8	80	10.5	
120,001-150,000 NTD	28	6.4	82	10.8	
≧ 15,0001 NTD	71	16.3	125	16.4	
Missing data	5	1.2	9	1.2	
Marital status					< .001
Single	108	24.8	178	23.3	
Married	259	59.5	409	53.6	
Divorced/Separated	48	11.0	156	20.5	
Widowed	20	4.6	19	2.5	
Missing data	0	0.0	1	0.1	
Living status					0.735
Live with spouse only	59	13.6	114	14.9	
Live with children only	81	18.6	162	21.2	
Live with spouse and children	138	31.7	225	29.5	
Live alone	42	9.7	71	9.3	
Other	115	26.4	191	25.0	
Medical history					
Heart disease	79	18.2	142	18.6	0.847
Hypertension	120	27.6	197	25.8	0.505
Dialysis	2	0.5	11	1.4	0.115
Stroke	51	11.7	45	5.9	0.121
Peptic ulcer	39	9.0	73	9.6	0.731
Thyroid disease	42	9.7	36	4.7	< .001
Chronic kidney disease	9	2.1	13	1.7	0.651
Hyperlipidemia	16	3.7	32	4.2	0.662
Diabetes	88	20.2	141	18.5	0.459
Gynecology disease	3	0.7	8	1.1	0.531
Asthma/Emphysema	9	2.1	15	2.0	0.903
Urinary disease	7	1.6	8	1.1	0.401
Cancer	4	0.9	2	0.3	< .001
Chronic liver disease	20	4.6	42	5.5	0.496
Gout	19	4.4	17	2.2	0.037
Pneumonia	8	1.8	3	0.4	0.012
Mental or psychiatric disease	14	3.2	11	1.4	0.039
Immunity disease	3	0.7	12	1.6	0.186
Others	105	24.1	190	24.9	0.768

NTD: New Taiwan dollar; A.F.F.H: agricultural, forestry, fishery or husbandry.

### The link between the SMS intervention and medication adherence

Comparing results before and after the SMS intervention (Table 2) shows that 78.8% of the participants in the intervention group had a decreased (i.e., no delayed doses) incidence of delayed doses; this figure was 46.4% for the control group. The differences between the pretest and post-test were significant for both groups (P < 0.05). Regarding missed doses, 90.1% of the participants in the intervention group showed a decrease (i.e., no missed doses), whereas only 61.1% of the participants in the control group showed a decrease between the pretest and post-test (P < 0.05 for both groups). Evaluating improvement regarding decreases in the frequency of delayed doses showed that 91.5 and 67.3% of participants in the intervention and control groups, respectively, who previously experienced delayed doses, demonstrated medication adherence improvement. Regarding decreases in the frequency of missed doses, 96.0 and 84.7% of participants who previously experienced missed doses, in the intervention and control groups, respectively, demonstrated medication adherence improvement (results not shown in the table). These findings suggest that SMS medication reminders effectively decreased the incidence of delayed and missed doses and enhanced patient adherence to medication.

**Table 2:** Comparing the results before and after the SMS intervention

	**Variable**	**Before SMS intervention**		**After SMS intervention**		**Improvement**	**P-value**
		**N**	**%**	**N**	**%**	**%**	
							< .001^a^
**Intervention group**	**Delayed doses (Total)**	646	84.7^c^	137	18.0^c^	78.8	< .001^b^
	20 ~ 34 y/o	146	90.1	32	19.8	78.1	
	35 ~ 49 y/o	181	79.7	52	22.9	71.3	
	50 ~ 64 y/o	196	85.6	35	15.2	82.2	
	≧ 65 y/o	123	84.8	18	12.4	85.4	
**Control group**	**Delayed doses (Total)**	349	80.2^c^	187	43.0^c^	46.4	< .001^b^
	20 ~ 34 y/o	83	86.5	58	60.4	30.1	
	35 ~ 49 y/o	105	77.8	62	45.9	41.0	
	50 ~ 64 y/o	113	77.4	54	37.0	52.2	
	≧ 65 y/o	48	82.8	13	22.4	72.9	
**Intervention group**	**Missed doses (Total)**	352	46.1^c^	35	4.6^c^	90.1	< .001^b^
	20 ~ 34 y/o	90	55.6	9	5.6	90.0	
	35 ~ 49 y/o	97	42.7	9	4.0	90.7	
	50 ~ 64 y/o	101	44.1	10	4.4	90.0	
	≧ 65 y/o	64	44.1	7	4.8	89.1	
**Control group**	**Missed doses (Total)**	190	43.7^c^	74	17.0^c^	61.1	< .001^b^
	20 ~ 34 y/o	52	54.2	28	29.2	46.2	
	35 ~ 49 y/o	62	45.9	14	10.4	77.4	
	50 ~ 64 y/o	52	35.6	30	20.6	42.3	
	≧ 65 y/o	24	41.4	2	3.5	91.7	

a: used chi-square test to examine the difference between groups; b: Used the McNemar’s test to examine the difference between the pre-test and post-test data within the group. c: intervention group% = n/763; control group%=n/435.

### Factors that influence decreases in the incidence of delayed doses

We used weighted logistic regression to identify the factors that affected the decrease in the incidence of delayed doses. Table 3 shows that participants in the intervention group had a 3.2 times (95% CI = 2.7–3.7) higher chance of a decrease in the incidence of delayed doses compared with participants in the control group.

**Table 3:** Weighted logistic regression of SMS on decreasing delayed doses

**Variable**	**OR**			**95% CI**	**P-value**
**After SMS intervention**					
No (reference)					
Yes	3.2	**	2.7	3.7	< .001
**Socioeconomic status**					
Gender					
Male (reference)					
Female	0.9		0.7	1.1	0.197
Age (years)					
20 ~ 34 (reference)					
35 ~ 49	0.6	**	0.4	0.8	0.001
50 ~ 64	0.9		0.6	1.2	0.419
≧ 65	1.0		0.6	1.5	0.955
Education level					
Elementary or less (reference)					
Junior High School	1.1		0.7	1.7	0.641
High School	2.1	**	1.4	3.2	< .001
College	1.3		0.8	1.9	0.288
University	0.9		0.6	1.5	0.782
Graduate school or above	0.6		0.4	1.1	0.076
Occupation					
Unemployed (reference)					
Military	1.4		0.4	5.3	0.582
Civil servant	0.9		0.5	1.4	0.579
Teacher	2.1	*	1.2	3.6	0.011
Student	1.0		0.6	1.7	0.938
Housekeeper	0.6	*	0.4	0.9	0.023
Self-employed	0.9		0.6	1.4	0.611
Medicine	1.8		1.0	3.5	0.064
Retired	0.7		0.4	1.1	0.145
Freelancer	0.7		0.4	1.2	0.234
Services	1.2		0.8	1.8	0.418
Worker	0.7		0.4	1.1	0.078
A.F.F.H.	2.7		0.8	9.7	0.127
Other	1.1		0.5	2.1	0.881
≦ 30,000 NTD (reference)					
30,001-60,000 NTD	0.8	*	0.6	1.0	0.043
60,001-90,000 NTD	1.6	**	1.2	2.1	0.001
90,001-120,000 NTD	0.8		0.6	1.2	0.254
120,001-150,000 NTD	0.7		0.5	1.0	0.062
≧ 150,001 NTD	2.1	**	1.5	3.0	< .001
Marital status					
Single (reference)					
Married	1.7	**	1.2	2.4	0.005
Divorced/Separated	0.7		0.5	1.1	0.143
Widowed	0.7		0.4	1.2	0.231
Living status					
Live with spouse only(reference)					
Live with children only	2.7	**	1.8	4.1	< .001
Live with spouse and children	0.7	**	0.5	0.9	0.006
Live alone	0.7	*	0.4	1.0	0.049
Other	1.3		0.9	1.9	0.149
Medical history					
Heart disease	0.7	**	0.5	0.9	0.001
Hypertension	0.7	**	0.6	0.9	0.001
Dialysis	0.6		0.3	1.3	0.217
Stroke	0.5	**	0.4	0.7	< .001
Peptic ulcer	1.8	**	1.2	2.5	0.002
Thyroid disease	0.6	**	0.4	0.8	0.001
Chronic kidney disease	0.7		0.4	1.3	0.285
Hyperlipidemia	1.0		0.7	1.5	0.876
Diabetes	1.0		0.8	1.3	0.930
Gynecology disease	0.9		0.4	1.8	0.672
Asthma/Emphysema	0.8		0.4	1.3	0.328
Urinary disease	1.0		0.5	2.0	0.992
Cancer	3.9	*	1.0	14.8	0.046
Chronic liver disease	1.3		0.9	2.1	0.199
Gout	1.3		0.8	2.1	0.357
Pneumonia	2.5		0.9	7.0	0.090
Mental or psychiatric disease	1.0		0.6	1.6	0.884
Immunity disease	3.9	**	1.67	9.0	0.002
Others	0.7	**	0.52	0.8	< .001

N = 1198. Dependent variable: 1 indicates with complete improvement; 0 indicates without complete improvement. Medical history reference group is the participants without this disease; A.F.F.H: agricultural, forestry, fishery or husbandry. * p < 0.05; ** p < 0.01.

Regarding medical history, participants with heart disease, hypertension, stroke, and thyroid disorder were less likely to have a decreased incidence of delayed doses compared with participants who lacked such diseases. However, participants with peptic ulcers, cancer, or immunological diseases were significantly more likely to experience a decrease in the incidence of delayed doses compared with participants who lacked such diseases.

### Factors that influence reduced incidence of missed doses

The weighted logistic regression results for factors that influenced the decrease in the incidence of missed doses showed that participants in the intervention group had a 2.2-fold (95% CI = 1.9–2.6) higher likelihood of experiencing a decrease compared with participants in the control group (Table 4). Additionally, women were significantly more likely to demonstrate improvement compared with men (OR = 2.1; 95% CI = 1.8–2.6). Participants with heart disease, hypertension, stroke, peptic ulcers, and thyroid disorder were less likely to show a decrease in the incidence of missed doses compared with participants who lacked such diseases. However, participants with psychiatric disorders (OR = 2.2, 95% CI = 1.3–3.8) were more likely to have a decrease in the incidence of delayed doses compared with participants who lacked such disorders.

**Table 4:** Weighted logistic regression of SMS on decreasing missed doses

**Variable**	**OR**		**95%**	**CI**	**P-value**
**After SMS intervention**					
No (reference)					
Yes	2.2	**	1.9	2.6	< .001
**Socioeconomic status**					
Gender					
Male (reference)					
Female	2.1	**	1.8	2.6	< .001
Age (years)					
20~34 (reference)					
35~49	0.7	*	0.5	1.0	0.024
50~64	0.7	*	0.5	0.9	0.014
≧ 65	0.6	**	0.4	0.9	0.009
Education level					
Elementary or less (reference)					
Junior High School	1.3		0.8	2.0	0.244
High School	0.7		0.5	1.1	0.138
College	0.7		0.5	1.1	0.100
University	0.7		0.4	1.0	0.058
Graduate school or above	0.6	*	0.4	1.0	0.034
Occupation					
Unemployed (reference)					
Military	0.1	**	0.0	0.3	< .001
Civil servant	0.4	**	0.2	0.6	< .001
Teacher	0.4	**	0.2	0.7	< .001
Student	0.5	**	0.3	0.8	0.005
Housekeeper	0.7		0.4	1.0	0.062
Self-employed	0.7		0.5	1.1	0.136
Medicine	0.7		0.4	1.3	0.277
Retired	0.7		0.5	1.1	0.164
Freelancer	0.9		0.6	1.5	0.711
Services	0.9		0.6	1.4	0.753
Worker	1.3		0.8	2.0	0.278
A.F.F.H.	2.3		0.7	7.9	0.190
Other	1.1		0.6	2.1	0.868
Average monthly household income					
≦ 30,000 NTD(reference)					
30,001-60,000 NTD	1.2		1.0	1.5	0.099
60,001-90,000 NTD	1.5	**	1.1	1.9	0.006
90,001-120,000 NTD	1.1		0.8	1.5	0.668
120,001-150,000 NTD	2.2	**	1.5	3.2	< .001
≧ 150,001 NTD	4.8	**	3.4	6.8	< .001
Marital status					
Single (reference)					
Married	1.4		1.0	1.9	0.065
Divorced/Separated	0.4	**	0.3	0.6	< .001
Widowed	1.7		1.0	2.9	0.054
Living status					
Live with spouse only (reference)					
Medical history	0.7		0.5	1.1	0.093
Live with spouse and children	1.4	*	1.1	1.8	0.013
Live alone	1.4		0.9	2.1	0.118
Other	0.6	**	0.4	0.8	0.004
Medical history					
Heart disease	0.4	**	0.3	0.5	< .001
Hypertension	0.4	**	0.4	0.5	< .001
Dialysis	0.5		0.2	1.0	0.051
Stroke	0.6	**	0.4	0.8	0.002
Peptic ulcer	0.7	*	0.5	0.9	0.014
Thyroid disease	0.7	*	0.5	1.0	0.028
Chronic kidney disease	0.7		0.4	1.3	0.252
Hyperlipidemia	0.8		0.5	1.1	0.186
Diabetes	0.9		0.7	1.1	0.354
Gynecology disease	1.0		0.5	2.2	0.965
Asthma/Emphysema	1.0		0.6	1.8	0.917
Urinary disease	1.0		0.5	2.2	0.910
Cancer	1.1		0.4	3.3	0.893
Chronic liver disease	1.4		0.9	2.0	0.111
Gout	1.5		0.9	2.3	0.089
Pneumonia	1.9		0.7	5.2	0.185
Mental or psychiatric disease	2.2	**	1.3	3.8	0.003
Others	0.5	**	0.4	0.6	< .001

N = 1,198; Dependent variable: 1 indicates with complete improvement; 0 indicates without complete improvement. Medical history reference group is the participants without this disease. The patients with immunity disease had no decrease on missed doses; A.F.F.H: agricultural, forestry, fishery or husbandry. * p < 0.05; ** p < 0.01.

### Impression of and satisfaction with the SMS intervention

Table 5 shows participants’ satisfaction with the SMS intervention; the mean satisfaction score was 4.3 out of 5. All satisfaction items scored at least 3 points, and the items “precision of wording,” “understandability of the content,” and “medication use privacy” scored more than 4 points. Regarding participant demand for the SMS intervention, “clearly displaying the medication dose” received the highest score, followed by “clearly displaying the frequency of dose.”

**Table 5:** Satisfaction with and demand for the SMS content

**Satisfaction with SMS content (N = 763)**	**Weighted mean**	**SD**
The SMS clearly describes the frequency of medication use.	3.8	0.7
The SMS clearly describes the method of medication use.	3.8	0.7
Frequency of SMSs received	3.1	1.0
Satisfaction with the precision of wording in the SMS	4.2	0.9
Satisfaction with the understandability of the content of the SMS	4.2	0.8
Satisfaction with medication use privacy in the SMS	4.2	0.9
Overall satisfaction with the SMS	4.3	0.7
**Demand for SMS content (N = 763)**	**Weighted mean**	**SD**
Demand for SMS clearly displaying the frequency of medication	2.8	1.0
Demand for SMS clearly showing method of medication	2.5	0.9
Demand for SMS clearly displaying the medication dose	2.9	0.9
Frequency of SMSs received	2.7	0.9

SMS: Short message service.

A 5-point Likert scale, ranging from 1, *not at all necessary/extremely unsatisfied*, to 5, *highly necessary/extremely satisfied*.

Of the participants who received SMS reminders (Table 6), 93.7% of the participants considered it unnecessary for a reminder to be resent after the time at which the medication should have been consumed. Additionally, 91.6% of participants in the intervention group reported a willingness to recommend the SMS service to their family and friends. In the intervention group, 83.1% of participants believed that the SMS intervention was helpful for preventing missed or delayed doses, and 73.7% of participants considered the service beneficial for disease management. However, 7.1% (N = 54) of participants indicated that the SMS did not help reduce delayed or missed doses; thus, we performed a subgroup analysis and determined that most of these participants were women (N = 39, 72.2%), married, and housekeepers.

**Table 6:** Demand for SMS reminders

**Variable**	**N = 763**	**%**	**Variable**	**N = 763**	**%**
**Preferring time to receive text messages before medication taken**			**Helpfulness of the text message medication reminder in improving the incidence of delayed or missed doses**		
10 minutes	262	34.3	Very unhelpful	13	1.7
15 minutes	65	8.5	Unhelpful	41	5.4
30 minutes	337	44.2	Neutral	75	9.8
60 minutes	71	9.3	Helpful	401	52.6
others	28	3.7	Very helpful	233	30.5
**Necessity of receiving text messages after the time medication should be consumed**			**Benefits of text message medication reminders for disease control**		
Yes	48	6.3	Very unhelpful	14	1.8
No	715	93.7	Unhelpful	55	7.2
**Willingness to recommend the text message service to family and friends**			Neutral	132	17.3
Yes	699	91.6	Helpful	373	48.9
No	64	8.4	Very helpful	189	24.8

## Discussion

The study intervention involved using SMS to remind patients to consume their medication. We evaluated patients’ medication adherence behavior and satisfaction with and demand for an SMS reminder service after the intervention. The SMS intervention significantly decreased the incidence of delayed and missed doses among participants in the intervention group, who showed a 29.0% decrease in the incidence regarding missed doses and 32.4% for the decrease in the incidence of delayed doses. These findings were similar to those reported by da Costa [8], who evaluated the effectiveness of and patient satisfaction with an SMS service that reminded women in Brazil who were diagnosed with AIDS to consume anti-viral drugs. Other studies have reported similar results, indicating that SMS reminders help patients consume their medications on time and reduce the incidence of delayed doses [12,13].

In the current study, the rates for the decreases in the incidence of delayed doses for the participants in the control and intervention groups were 46.4 and 78.8%, respectively; these figures were 61.1 and 90.1%, respectively, for decreases in the incidence of missed doses. This indicates that participants in the control group also experienced significant improvement in medication adherence. This could result from the Hawthorne effect or increased efficacy in self-managing their illnesses [14]. Previous studies have shown that in the healthcare field text message reminders were effective in increasing patient attention to treatment, decreasing the incidence of missed doses, and enhancing medication adherence [8,15].

We followed up with participants after only 7 days; however, long-term adherence is more difficult to maintain than is short-term adherence. Hanauer et al. used e-mail and SMS reminders to support diabetes management and identified decreases in SMS use after 2 months and 3 months [16]. Another study of SMS in adults with diabetes showed no change in blood glucose measurement activity throughout the 1-year study period [17]. Therefore, SMS messaging may be more appropriate for use with medications, such as some antibiotics, taken in the short term, compared with long-term medications used to treat chronic diseases.

### SMS improves medication adherence

We used weighted logistic regression to examine the factors that influence improvement in the incidence of delayed and missed medication doses. The results showed that the SMS intervention significantly decreased the incidence of delayed and missed doses. This was consistent with a previous finding that indicated SMS interventions enabled patients to consume their medication on time [18]. Regarding age (Table 4), participants aged 65 years or older were significantly less likely to experience decreases in the incidence of missed doses compared with those aged 20–34 years (OR = 0.6, 95% CI = 0.4–0.9). According to related literature, senior patients are often more resistant to behavioral change and are likely to stop taking medication based on personal decisions [19]. In addition, senior patient may be less familiar with cell phones compared with younger patients, increasing their likelihood of ignoring text message reminders, thus limiting decreases in the incidence of missed doses. However, comparing Tables 3 and 4 shows that when senior participants neglected or delayed taking medication, after they received SMS reminders they had a higher likelihood of taking medication than did those who missed taking the medication. This suggests that if senior participants delayed taking medication, when they received reminders they had a higher likelihood of improving their adherence than did those who lacked a strong intention to take their medication and missed taking it despite the reminder. Military personnel, civil servants, teachers, and students demonstrated similar behaviors. These results were similar with those of a previous study [20].

Regarding occupation, military personnel, civil servants, teachers, and students were less likely to experience decreases in the incidence of missed doses compared with unemployed participants. This could be because the military personnel, civil servants, teachers, and students in our study were typically young or middle-aged adults who demonstrated relatively better health compared with unemployed participants; therefore, these participants might consider long-term medication use unnecessary.

Regarding medical history, participants with hypertension, heart disease, stroke, or thyroid disease were less likely to experience decreases in the incidence of delayed or missed doses compared with participants who lacked such diseases. This finding was consistent with that reported by a previous study [20]. This study indicated that hypertensive patients tended to have confidence in their ability at self-control and were likely to adjust their medication consumption behavior arbitrarily, leading to poor adherence [20]. Therefore, SMS interventions may not reduce the incidence of delayed or missed doses among specific patient groups. A previous study also emphasized that patients with chronic diseases may have less motivation to consume medication regularly if their condition did not seem to improve, was incurable, or yielded side effects because of long-term medication use [21]. Thus, SMS interventions may fail to significantly alter the medication consumption behavior of patients with chronic diseases.

### Satisfaction with and demand for the SMS intervention

Numerous observational studies have focused on satisfaction with and demand for SMS interventions [22-25]. In the current study, according to the results of participants’ satisfaction with the SMS intervention, the item “frequency of the SMS” received the lowest satisfaction score of 3.1 points. Participants reported that “the frequency of text messages sent was too high,” “text messages did not need to be sent often,” and “the number of messages was excessive.” These responses indicate that although the SMS intervention could help remind participants to consume medicines, it could also induce negative perceptions. Regarding participant demand for the SMS intervention, most participants (44.2%) wished to receive a reminder 30 minutes before the time medication should be consumed (Table 5). However, 93.2% of participants disliked when a message was resent after the time medication should be consumed to ensure that they would immediately consume a missed dose. Approximately 91.6% of the participants were willing to recommend the SMS intervention to their family and friends, suggesting that the SMS reminder intervention was effective. Of the participants who stated that the SMS reminders did not reduce delays or missed doses, most were women (72.2%), married, and housekeepers. We speculate that because these participants had more flexible time to care for themselves and may already have been adhering to their medication satisfactorily, they did not perceive that the SMS intervention enhanced their medication adherence.

Previous studies that have adopted SMS interventions for increasing medication adherence all showed that patients in the intervention group experienced higher treatment effectiveness compared with control groups. This could be because SMSs pose a minimal interruption to patients’ lives and are low in cost [8]. Compared with other approaches, SMS is simpler and more satisfying for users. Additionally, because the SMS reminder service can prompt patients to be responsible for their own health, it serves a vital function in healthcare services.

### Limitations

Medication adherence problems are typically related to use of long-term medications for chronic diseases. In the current study, participants were monitored for only 7 days. Such a short-term follow-up might not properly interrogate the relationship between long-term medication adherence and use of SMS. Because we did not design the study to specify the medications used by participants, we could not evaluate the effects and outcome of their medication treatment. Additionally, the daily SMS system sent more than one reminder to participants who took several kinds of medications based on various medication schedules, which resulted in inconveniencing these participants. Many studies have shown that medication adherence outcome data that are purely reliant on self-reporting have a high likelihood of reporting bias [26,27]. Because overstimulation might exist in both the intervention and control groups, the current study included two groups (intervention vs. control) that completed pretests and post-tests to reduce the effects of reporting bias.

## Conclusions

This controlled study showed that SMS intervention enhanced patients’ medication adherence. After the 7-day SMS intervention, patients in the experimental group showed greater decreases in the incidences of both delayed and missed doses compared with the control group patients. The findings of this study and those of previous studies show that use of SMS can effectively improve patient medication adherence, prompting most patients to respond favorably to such services. Therefore, an SMS reminder system is a simple, effective, and inexpensive strategy [28,29]. Future implementation of SMS in Chinese-language settings could contain pictures and both Chinese and English drugs names, enabling patients to verify their use, thereby decreasing the incidence of delayed and missed doses.

## Abbreviations

SMS: Short message service; PMP: Personal medication management platform; WHO: World Health Organization; HIS: Hospital information system; NTD: New TAIWAN Dollar; AIDS: Acquired immunodeficiency syndrome.

## Competing interests

The authors declare that they have no competing interests.

## Authors’ contributions

HLH and WCT drafted the manuscript. WCT and YCL designed the study. YCC, YWH, FK, PTK and CJC collected data. SDC, PTK and BYJL were responsible for study conceptualization and developing the analytical plan. All authors read and approved the final manuscript.

## Pre-publication history

The pre-publication history for this paper can be accessed here:

http://www.biomedcentral.com/1472-6947/13/127/prepub
